# Surgical site infections following transcatheter apical aortic valve implantation: incidence and management

**DOI:** 10.1186/1749-8090-7-122

**Published:** 2012-11-13

**Authors:** Richard Baillot, Éric Fréchette, Daniel Cloutier, Josep Rodès-Cabau, Daniel Doyle, Éric Charbonneau, Siamak Mohammadi, Éric Dumont

**Affiliations:** 1 Department of Cardiac Surgery, Université Laval, Québec City, Qc, Canada; 2 Division of Thoracic Surgery, Université Laval, Québec City, Qc, Canada; 3 Department of Plastic Surgery, Université Laval, Québec city, Qc, Canada; 4 Department of Cardiology, Université Laval, Québec city, Qc, Canada

**Keywords:** Transapical aortic valvular implantation (TA-TAVI), Surgical site infection (SSI)

## Abstract

**Objective:**

The present study was undertaken to examine the incidence and management of surgical site infection (SSI) in patients submitted to transapical transcatheter aortic valve implantation (TA-TAVI).

**Methods:**

From April 2007 to December 2011, **154** patients underwent TA-TAVI with an Edwards Sapien bioprosthesis (ES) at the Institut Universitaire de Cardiologie et Pneumologie de Québec (IUCPQ) as part of a multidisciplinary program to prospectively evaluate percutaneous aortic valve implantation. Patient demographics, perioperative variables, and postoperative complications were recorded in a prospective registry.

**Results:**

**Five (3.2%)** patients in the cohort presented with an SSI during the study period. The infections were all hospital-acquired (HAI) and were considered as organ/space SSI’s based on Center for Disease Control criteria (CDC). Within the first few weeks of the initial procedure, these patients presented with an abscess or chronic draining sinus in the left thoracotomy incision and were re-operated. The infection spread to the apex of the left ventricle in all cases where pledgeted mattress sutures could be seen during debridement. Patients received multiple antibiotic regimens without success until the wound was surgically debrided and covered with viable tissue. The greater omentum was used in three patients and the pectoralis major muscle in the other two. None of the patients died or had a recurrent infection. Three of the patients were infected with *Staphylococcus epidermidis*, one with *Staphylococcus aureus*, and one with *Enterobacter cloacae*. Patients with surgical site infections were significantly more obese with higher BMI **(31.4±3.1 vs 26.2±4.4 p=0.0099)** than the other patients in the cohort.

**Conclusions:**

While TA-TAVI is a minimally invasive technique, SSIs, which are associated with obesity, remain a concern. Debridement and rib resection followed by wound coverage with the greater omentum and/or the pectoralis major muscle were used successfully in these patients.

## Background

The cardiac surgery community has recently suggested that per-cutaneous aortic valve replacement be used to replace diseased aortic valves in an aging population with significant co-morbidities. Current results as well as early event-free survival with this new minimally invasive surgical technique are encouraging [1].

However, complications can occur [2-9] and the explicit indications and limitations of this technique are evolving due to the relatively small number of case series published to date [9]. The work reported here focussed on the incidence, management, and outcome of surgical site infectons (SSI’s) in patients undergoing trans-apical aortic valvular replacement (TA-TAVI) at IUCPQ.

## Methods

The present study was a single-centre prospective case series. One hundred and fifty four consecutive patients who underwent TA-TAVI with an Edwards Sapien (Edwards Lifesciences, Irvine, CA, USA) bioprosthesis between April 2007 and December 2011 were included in this retrospective analysis of prospectively collected data. Patient demographics, perioperative variables, and postoperative complications were prospectively recorded in a dedicated registry. Data collection procedures complied with the ethical rules of our research centre, and informed consent was obtained from the patients prior to surgery.

### Surgical technique

The TA-TAVI technique has been described in a previous publication [10] and by our institute and others in Europe in the Source registry. Briefly, the procedure is performed on heparinized patients (ACT 250 s) in a surgical theatre with fluoroscopy and under trans-oesophageal guidance with full monitoring. A small left anterolateral mini-thoracotomy is usually performed in the fifth intercostal space aligned on the mid-clavicular line. Pledgeted pursestring 2–0 Ethibond sutures are applied on the left ventricular apex (LV) after dissection and pericardial opening. A 26 Fr sheath is then inserted in the left ventricular apex and a 23 or 26 mm ES prosthesis is inserted under rapid pacing, trans-oesophageal guidance, and fluoroscopy [11]. Haemostasis is then performed again under rapid pacing to avoid tension and tearing of the ventricle. The pericardium is re-approximated whenever feasible. Conversion to extracorporeal bypass was needed in six (5.3%) patients in our cohort.

### Statistical analysis

Continuous variables are reported as means ± standard deviation, or as medians ± interquartile range, when appropriate. Discrete variables are reported as percentages unless otherwise specified. Factors associated with SSIs were analyzed using the chi-square test and Fisher’s exact test, when appropriate. Because of the low number of outcomes, a multivariable analysis was not performed. All tests were two-tailed, and a *P* <0.05 was considered statistically significant. Analyses were conducted using Jump Software, v.7.0.2 (copyright 2007, SAS Institute Inc.).

## Results

One hundred and fifty-four patients were recruited for the study. The mean age of the patients was 78.6±7.9, and 57.8% were women. Previous cardiac surgery was documented in 70 (45.5%) of the patients, and 115 (74.7%) had coronary artery disease. Chronic renal failure was the rule in these aging patients, and the mean creatinine clearance rate (Cockroft formula) was 39.7±16.4 cc/min. The men and women had similar BMIs (M: 26.9±5.1/W: 26.8±5.5).

Five (3.2%) of the patients developed an SSI during the study period. Patient demographics are listed in Table 1. The mean age of these five patients with infected wounds was 75±6.3 and four were women. All were hypertensive and dyslipidemic and the four women presented with morbid obesity (BMI>30). One patient was formally identified as being diabetic, and three presented with hypothyroidism. Two patients had significant COPD with an FEV1 <70%. Two patients also presented with peripheral vascular disease while two others had a history of deep venous thrombosis and/or pulmonary emboli. All five patients who developed an SSI presented with significant chronic renal failure, three of whom having a creatinine clearances <60 cc/min. Lastly, one patient had been treated by radiotherapy for a lung carcinoma in the past, with resultant pulmonary fibrosis, while another was being treated with steroids for rheumatoid arthritis.

**Table 1:** Demographics of TA-TAVI hospital-acquired infections

**Patient**	**Sex**	**Age**	**LV%**	**KG/BMI**	**Co-morbidity**	**ES**	**Complications**
1. AB	F	79	65	66/33	TIA	23	Pneumothorax
					Hypothyroidism		UTI
					Previous CABG		CK-MB: 32.4
					Cr cl: 31.0		
2. JGC	M	79	50	78/27	AF/COPD	26	AF, renal failure,
					PVD		heart failure, pneumonia, empyema CK-MB: 33.4
					Gout		
					RA/steroids		
					History DVT Previous CABG		
					Cr cl: 31.8		
3. IR	F	76	65	72/30	Hypothyroidism	23	AF/Flutter
					Previous DVT/PE		CK-MB: 18.8
					Porcelain aorta		
					Cr cl: 61.8		
4.MB	F	64	50	92.5/35.2	DM/MI	26	Heart failure
					Pulmonary emboli		CK-MB: 12.4
					Lung Ca/Rorx		
					Cr cl: 69.4		
5. CR	F	77	20	82/31.2	PAF/PVD	26	Heart failure
					Hypothyroidism		CK-MB : 12.4
					Active smoker		
					COPD		
					Heart failure		
					Cr cl: 26.5		

*AF* Atrial fibrillation, *CA* cancer; *CABG*; Coronary artery bypass grafting; *Cr cl,* Creatinine (clearance ml/min); *COPD* Chronic obstructive pulmonary disease; *DVT* Deep venous thrombosis; *DM* Diabetes mellitus; *MI* Myocardial infarction; *PAF*, Paroxysmal AF; *PE* Pulmonary emboli; *RA* Rheumatoid arthritis; *RoRX* radiotherapy.

The indications for TA-TAVI in all five patients were severe senile degenerative calcified aortic stenosis (≤0.8cm) with a documented porcelain aorta in one patient and significant co-morbid conditions that would normally have denied them a standard aortic valvular replacement by sternotomy with extracorporeal bypass. All were NYHA class III, and only one patient presented with severe LV dysfunction with a history of heart failure, while two had had a coronary artery bypass.

### Surgical site infections: presentation and diagnosis

**Figure 1:**
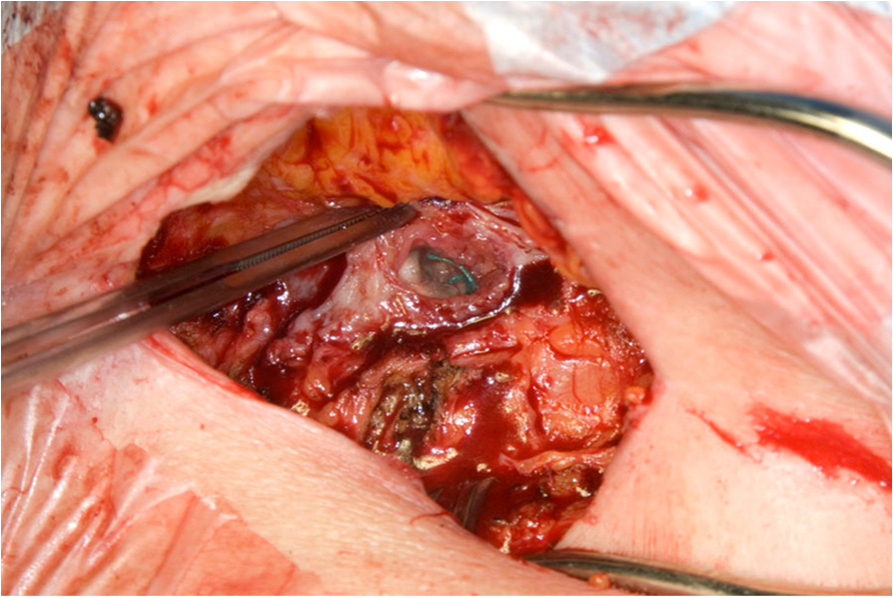
Chronic sinus leading to left ventricular apex.

All five infections were hospital-acquired (HAI), that is, they occurred within one year of surgery, and were classified as organ/space SSIs based on CDC criteria. One patient also developed empyema. No infections were associated with post-implant prosthetic valve endocarditis. All five patients presented with an abscess or a chronic draining sinus in the left thoracotomy incision within the first few weeks of the initial procedure. In all cases, the infections spread to the apex of the left ventricle where pledgeted mattress sutures could be seen during re-exploration and debridement. This site was the previous port of entry for the TA-TAVI procedure. (Figure 1) None of these patients presented with a false aneurysm of the left ventricular apex or septic shock.

### Microbiology, surgical management, and outcome

All patients were treated using a combination of antibiotherapy and surgical debridement. As a rule, the patients received multiple antibiotic regimens with no success until the wound was surgically debrided and covered with viable tissue (greater omentum and/or pectoralis major muscle). Three patients had a *Staphylococcus epidermidis* infection, one a *Staphylococcus aureus* infection, and the fifth an *Enterobacter cloacae* infection. With more than a year of follow-up for all patients, none of the patients has died or presented with a recurring infection.

### Factors associated with SSIs

Univariate analyses were performed to identify factors associated with an increased risk of infection. When compared to the other patients without infections, these five patients were found to be more obese (BMI >30) and did present with chronic renal failure. (Table 2) No other factors with a significant correlation with an increased risk of infection were found.

**Table 2:** TA-TAVI infection vs no-infection demographic and operative variables

	**No infection**	**Infection**	***P***
	**(n=149)**	**(n=5)**	
Age	78.7±7.9	75±6.3	0.3079
Men (%)	43.0 (64/149)	20 (1/5)	0.3067
Weight (kg)	67.1±14.9	78.2±10	0.0996
BMI	26.7±5.3	31.4±3.1	0.0504
HBP (%)	82.6 (123/149)	100 (5/5)	0.3056
DM (%)	34.2 (51/149)	20 (1/5)	0.5081
Cr.clearance (median)	39.7±16.5 (37.5)	44.1±19.9 (31.8)	0.5580
DLP (%)	89.9 (134/149)	100 (5/5)	0.4552
PVD (%)	45.6 (68/149)	40 (2/5)	0.8033
COPD (%)	26.2 (39/149)	40 (2/5)	0.4915
Stroke (%)	3.4 (5/149)	0 (0/5)	0.6771
GI comp (%)	6.7 (10/149)	20 (1/5)	0.2564
ARF (%)	18.1 (27/149)	20 (1/5)	0.9147
Septicaemia (%)	0	0	-
Bronchitis/Pneumonia (%)	21.5 (32/149)	40 (2/5)	0.3260
UTI (%)	18.1 (27/149)	20 (1/5)	0.9147
Death (%)	12.8 (19/149)	0	0.3938

## Case presentation

### Patient 1

AB was a 81-year-old patient who had undergone coronary artery bypass graft surgery (CABG) twice in the past (1973 and 1993). She underwent a TA-TAVI procedure on March 26, 2008, with an ES 23, which was followed by a chronic infection of the thoracic incision. Cultures revealed the presence of *E. cloacae*. AB was followed at another centre and was referred back to us for surgery approximately two years after the initial surgery. The wound was debrided, and two adjacent segments of rib were resected and shaved from the left ventricule (LV) apex where Ethibond® pledgeted sutures could be seen. The LV apex was then covered with the inferior part of the pectoralis major muscle, which was easily mobilized. A seroma was drained a few days later, and the wound subsequently healed without complication.

### Patient 2

JGC was a 79-year-old patient who presented with rheumatoid arthritis and a history of previous CABG. He underwent the TA-TAVI procedure with an ES 26 without serious complications on June 2, 2009. Transient heart failure and an SSI occurred one month later. Cultures were positive for *S. epidermidis*. JGC was re-operated following drainage and local debridement, and part of the fifth rib was resected and a left lower lobe empyema was decorticated. The LV apex was covered with a pectoralis major muscle flap after complete mobilization that included humeral detachment to obtain an adequate amount of vascularized tissue to cover the LV apex and fill the adjacent residual thoracic cavity. This patient did well, and a Gallium scan performed four months later showed no abnormal captation.

### Patient 3

IR was a 76-year-old patient who underwent a TA-TAVI procedure with an ES 23 on June 2, 2009. Severe calcification of the ascending aorta was identified pre-operatively, and IR was referred for a percutaneous aortic valve implantation. The procedure was uneventful but the patient later presented with an *S. epidermidis* SSI. Despite adequate antibiotherapy, continuous drainage from the wound was documented, and the patient was referred for debridement and surgery four months after the initial surgery. Following an upper midline mini-laparotomy incision, the greater omentum was mobilized and brought to the left hypochondrium to cover the LV apex after thorough debridement of the incision.

Mobilization of the pectoralis was avoided in this patient due to the presence of huge breasts and fear of glandular necrosis. Follow-up was uneventful, and a Gallium scan performed two months later showed no abnormal isotopic fixation. This patient later presented with an incisional hernia and was reoperated.

### Patient 4

MB was a 64-year-old obese diabetic patient who underwent a TA-TAVI procedure with an ES 23 on June 2, 2009. MB came back two months after the surgery, and an abscess in the thoracic incision was drained. Cultures revealed the presence of *S. aureus*. The past medical history of this patient included a right upper lobe adenocarcinoma treated with radiotherapy and subsequent pulmonary fibrosis. This patient had a history of pulmonary emboli.

Despite adequate antibiotherapy, continuous drainage from the wound was documented, and debridement was scheduled. Due to previous chest irradiation, an upper midline mini-laparotomy was performed at the same time. The greater omentum was mobilized, brought through a small incision, and tunnelled to the left hypochondrium to cover the LV apex where the pledgeted felts and sutures used for previous haemostasis could be seen. CT and Gallium scans performed three months after covering the LV apex with the greater omentum showed no signs of a recurring infection.

### Patient 5

CR was a 76-year-old patient who underwent a TA-TAVI with an ES 26 in October 2009. An *S. epidermidis* SSI ensued, which was locally debrided. The greater omentum was mobilized and used to cover the LV apex. However, the patient presented with a recurring infection, which was re-explored and further debrided. A rib resection over the LV apex was performed to complete the debridement. The lower part of the pectoralis major muscle was then partially mobilized and was used to cover the LV apex. A Gallium scan performed four months later showed no residual captation or signs of infection.

A review of all the operative protocols of these patients revealed that four of these five patients were operated in the first half or early experience of this series, that three were implanted the same day, and that the pericardium of only one infected patient was re-approximated during the initial procedure.

Haemostatic agents such as Cryolife-Bioglue® (purified bovine serum albumin and glutaraldehyde) and Ethicon-Knu-Knit® (sterile absorbable knitted fabric prepared by the controlled oxidation of regenerated cellulose) were also used in 37 (32.7%) patients in this series to help control bleeding from the ventricle. BioGlue was used in only one of these 5 patients with infection.

## Conclusion

Although numerous studies have reported low frequencies of SSIs in patients who have undergone cardiac surgery, the present study is the first to describe the infectious complications associated with TA-TAVI in detail as well as their management and outcome. SSIs remain one of the most important and dreaded surgical complications of all cardiac procedures. They can affect up to 2 to 5% of these patients and are the most frequent HAIs [12].

SSIs are a costly complication in all types of surgery and are associated with prolonged hospital stays, psychological distress, and increased mortality [13-16]. The commonly acknowledged risk factors are associated with the changing demographics of patients presenting with cardiovascular diseases and are linked to aging, obesity, diabetes, and chronic renal failure [17,18].

The same principles used to cure deep sternal wound infection (DSWI) were used in the present study, with viable tissues i.e. pectoralis muscle and/or greater omentum being brought to the wound to achieve complete healing.

Following wound washout and debridement of nonviable tissues, resection of a segment of the adjacent anterior part of the 5^th^ rib is recommend. Then and after partial mobilization of the inferior part of the pectoralis major, the LV apex is completely covered. In cases where further muscle tissue is needed, the pectoralis major can be completely mobilized from its insertions (including humeral) to optimize local treatments with minimal compromises in terms of postoperative function. Avoiding the use of the greater omentum may minimize abdominal complications such as herniation, although the omentum can now be easily mobilized with much less dissection using abdominal laparoscopic techniques.

Transposition of the greater omentum to reconstruct the chest wall through a subcutaneous tunnel has been described in the past to cure patients with complex skin ulceration following Halstead radical breast cancer surgery and chest wall irradiation [19] although Kiricuta [20] is mainly credited for the initial use of the greater omentum for chest wall reconstruction. The omentum has a known power of repair, is obviously very well vascularised and can regenerate tissue and cure infection [21].

Interestingly, we were able to cure all the infected patients despite the fact that foreign material was left behind i.e. apical pledgeted sutures. These patients have now all been followed for more than a year, and show no signs of recurring infections.

No severe sepsis, haemorrhaging, or false aneurisms were observed in these patients despite the fact that the left ventricular apex could be seen during surgical debridement and exposure. In our experience, the post-operative course of these patients was also uneventful, and the associated morbidity and mortality to date is lower than in patients afflicted with DSWI.

Pasic did report 3 patients that present late wound healing problems following TA-TAVI attributed to the use of glutaraldehyde and BioGlue [22]. In their publication they did report that LV apical sutures and felt were all removed from the LV apex without bleeding complications.

Preventive measures taken before the TA-TAVI procedure should be the same as with any other cardiac interventions with extracorporeal circulation. It include a dental consultation, eradication of bacteriuria, and prophylactic treatment of nasal carriers of *S. aureus* with mupirocin and chlorhexidine bathing if the patient is not an emergency. Antibioprophylaxis should also be pursued aggressively in due time before surgery for these patients who often present with lifelong sequelae of diabetes.

During surgery, glycaemic control is mandatory and pericardial closure is recommend, as in any other standard open heart surgery.

To our knowledge, there are still very few descriptions in the literature of the results and infectious complications of TA-TAVI. Bleiziffer et al. [23] reported that two of 50 TA-TAVI patients (4%) had secondary wound healing problems that were managed with negative pressure wound therapy and delayed primary wound closure. We didn’t use negative wound pressure therapy for these patients with an open sinus leading to the apex of the left ventricle. Pasic [7] reported that two of 175 TA-TAVI patients (1.1%) had post-operative wound problems, one of whom being an MRSA carrier who eventually died from sepsis and another one who died from a groin infection following conversion to femoro-femoral cardiopulmonary bypass.

Minimally invasive valvular surgery and transcatheter aortic valvular replacement have recently been put forward as alternatives to standard open valvular replacement via sternotomy. These techniques are associated obviously with better cosmetic results, less pain and blood loss, better respiratory function and shorter hospital stays. They are challenging the way valvular surgery has been performed in the past and, as suggested by L. Cohn, and are opening the way to a paradigm shift in cardiac surgery [24].

The percentage (3.2%) of SSIs seen in this cohort of TA-TAVI patients was within the range of 2 to 5% observed in clean surgery. The infections mostly occurred during the learning curve of this procedure in our centre or during the first half of the cohort submitted to TA-TAVI.

The present study is the first to describe in detail the infectious complications associated with TA-TAVI as well as their management and outcome with pectoralis myocutaneous flaps and greater omentum transposition. Overall, the infections following TA-TAVI were mainly caused by Gram-positive species that are also seen in patients with DSWI. However, they caused less morbidity than infections following sternotomy. While the present study was underpowered and observational, a higher BMI was found to be a significant predictors of SSIs in this cohort of patients who underwent this minimally invasive valvular procedure.

## Abbreviations

BMI: Body mass index (kg/m2); CABG: Coronary artery bypass graft; DSWI: Deep sternal wound infection; ES: Edwards Sapien valve; HAI: Hospital-acquired infection; IUCPQ: Institut Universitaire de Cardiologie et de Pneumologie de Québec; LV: Left ventricule; SSI: Surgical site infection; TA-TAVI: Transapical transcatheter - aortic valve implantation.

## Competing interests

None declare by the all the authors.

## Authors’ contributions

RB Main author Wrote the paper. EC Main co-author Wrote the paper. DC Main consultant in plastic surgery Re-read and comments on reconstructive techniques. JRC Comments on TAVI patients presenting with SSI's Author of many papers on percutaneous valve implantation from our tertiary center. DD Surgeon involve with TAVI program Re-read the paper and comments. EC Co-author Re-read and comments. SM Co-Author Re-read and comments. ED Surgeon involve with TAVI program RE-read and comments. All have reviewed the submitted manuscript and did approved the final draft.
